# Economic experiments support Ostrom’s polycentric approach to mitigating climate change

**DOI:** 10.1057/s41599-022-01436-6

**Published:** 2022-12-10

**Authors:** Manfred Milinski, Jochem Marotzke

**Affiliations:** 1 Max-Planck-Institute of Evolutionary Biology, Plön, Germany; 2 Max Planck Institute for Meteorology, Hamburg, Germany

**Keywords:** Social policy, Economics

## Abstract

The late Nobel laureate Elinor Ostrom envisioned a polycentric approach to mitigating climate change rather than a centralised solution. Debating about global efforts to solve climate-change problems has yet not led to an effective global treaty. Ostrom argued that instead of focusing only on global efforts, it is better to encourage polycentric efforts to reduce the risks associated with the emission of greenhouse gases. Many problems conceptualised as ‘global problems’ are the cumulative results of actions taken by individuals, families, small groups, private firms, and local, regional, and national governments. Ostrom and colleagues pointed to many examples of successfully managing a common good through interaction within a community. Energy-saving actions undertaken by individuals, families and actors at a small-scale pay off and, when multiplied, may reduce emissions globally. The incentive to achieve an individual net gain may trigger human investment decisions. Here we provide experimental support for Ostrom’s basic ideas using methods of experimental economics. By subdividing experimental populations in subgroups that approach sub-goals of mitigating simulated dangerous climate change combined with incentives, the ‘global’ solution is achieved by combined subgroup contributions exceeding the ‘global’ threshold for averting simulated dangerous climate change. Incentives from refunded saved energy motivate reaching sub-goals, as Ostrom suggested. By contrast, coercing free-riding subgroups through sanctioning at a cost fails, because sanctioning also hits fair individuals who then reduce their contributions. However, the power of polycentricity with numerous successful units can help mitigate climate change.

## Introduction

The global community of countries has entered the legally binding Paris Agreement (COP21) to limit global warming to well below 2 °C and to pursue efforts to limit warming to 1.5 °C. Achieving these two goals requires CO_2_ emissions to reach net-zero by the middle of the century or during the second half of the century, respectively (IPCC, 2021). Nevertheless, global CO_2_ emissions have kept rising even after the adoption of the Paris Agreement, apart from the dip caused by the COVID-19 pandemic (Friedlingstein et al., 2021). Current pledges by individual countries to reduce emissions are not putting the world on a path toward net-zero CO2 emissions (Hausfather and Peters, 2020), and a recent comprehensive assessment concluded that the current social dynamics toward decarbonisation are not powerful enough for zero CO_2_ emissions by 2050 (Stammer et al., 2021). A plausible explanation for the slow path toward decarbonisation is the tendency toward, and fear of, free-riding among the countries sending delegations to UN conferences (Milinski et al., 2016), ultimately an example of Hardin’s tragedy of the commons (Hardin, 1968).

However, Ostrom criticised (Ostrom et al., 1999; Berkes et al., 1989; Dietz et al., 2003) Hardin’s claimed inevitability that ‘whenever people have free access to a public resource, the resource will be overused and collapse’ (Hardin, 1968), and that resource users are trapped in a common’s dilemma, unable to create solutions, and thus can be rescued only by a centralised government. Instead Ostrom propagated a decentralised, ‘polycentric’ approach to mitigating global climate change (Ostrom, 2009a, 2009b; Ostrom, 2012; Ostrom, 2014). She and her co-workers (Dietz et al., 2003; Berkes et al., 1989; Ostrom, 1990; Ostrom et al., 1992; Vollan and Ostrom, 2010) referred to the many social groups that have struggled successfully against threats of resource degradation by developing and maintaining self-governing institutions. Many examples exist of long-term sustainable resource use in economically advanced communities with effective, local, self-governing rights, requiring that rules of resource use are generally followed based on trust but also by imposing modest sanctions (Dietz et al., 2003; Ostrom, 2009a; Ostrom, 1990). It is often true that relatively small groups in large societies, such as local communities, have enormous potential to organise and manage themselves in ways that promote cooperation and prevent them from depleting natural resources. These are important virtues of a local organisation, formal or informal, relative to a more global authority (Van Lange et al., 2013). Social bonding and social networks are favoured in small-scale societies (Boehm, 2019). By contrast, the efforts of an external authority to change the group behaviour in a certain direction can, counter-intuitively, have an opposite effect on individual behaviour (Gavrilets, 2021).

Vincent Ostrom (1999) defined ‘a polycentric order as one where many elements are capable of making mutual adjustments for ordering their relationships with one another within a general system of rules where each element acts with independence of other elements.’ ‘Polycentricity is a useful analytical approach for understanding and improving efforts to reduce the threat of climate change’ (Ostrom, 2010). Polycentric theory generally assumes that actors will mobilise against a problem when it is in their self-interest to do so (Jordan et al., 2018). Ostrom’s hypothesis builds on the human habit of being cooperative if it pays off through individual gains (Ostrom, 2010; Milinski et al., 2002; Milinski et al., 2006). She identifies this opportunity when families and small firms can achieve benefits that offset costs at a household or neighbourhood level or by small firms that invest in better construction of a building, investment in solar panels, and many other investments in equipment that families as well as private firms can make that pay off in the long run. ‘The important point is that benefits can be achieved that offset costs at the household or neighbourhood level‘ (Ostrom, 2012). ‘At least seventeen actions that can be taken within a home or a business facility can cumulatively have a major impact on carbon emissions and save the family or firm budget‘ (Dietz et al., 2009). The incentive to achieve this net gain, thereby reducing greenhouse gas emissions, may trigger human investment decisions. Even though each of these small units saves energy and thus CO_2_ emissions at a small scale, it is the multitude of these units that can make polycentricity successful at the global level (Ostrom, 2010, 2012, 2014).

Interestingly, the minister of trade and commerce of a European country has just suggested that all households together each saving 10% energy could resolve the country’s energy crisis—very close to Ostrom’s ideas.

’The emergence of a polycentric system is argued to start the process of reducing greenhouse gas emissions and to act as a spur to international regimes to do their part’ (Ostrom, 2012; Green et al., 2014; Jordan et al., 2015; Cole, 2015). Although Ostrom (2012) stresses that ‘researchers need to understand the strength of polycentric systems where enterprises at multiple levels may complement each other‘, she always comes back to mention ‘the importance of building a strong commitment to finding ways of reducing individual emissions for coping with climate change‘. The familiar slogan ‘Think globally but act locally’ hits right at the dilemma facing all inhabitants of the world: ‘To solve climate change in the long run, the day-to-day activities of individuals, families, firms, communities, and governments at multiple levels must change substantially‘ (Ostrom, 2010). ‘It is obviously much easier to craft solutions for collective-action problems related to smaller scale common pool resources than for the global commons‘ (Ostrom, 2009a).

Jordan et al. (2018) provide a comprehensive state-of-the-art discussion of the polycentric approach to climate governance, its promise and potential limits. However, while both self-governed stable preservation of common goods and successful multiscale governance demonstrably exist, experimental evidence has been lacking so far that indeed Ostrom’s polycentric approach is more successful at maintaining a global common good than relying on global agreements alone. It is this gap that we try to fill here. ‘We use the design of the ‘collective-risk social dilemma’ that arises from attempts to avert simulated dangerous climate change in experimental games‘ (Milinski et al., 2008; Milinski et al., 2011; Tavoni et al., 2011; Jacquet et al., 2013; Milinski et al., 2016; Dannenberg et al., 2015; Skatova et al., 2016; Kline et al., 2018; Andrews et al., 2018; Marotzke et al., 2020; Andrews et al., 2021; Szekely et al., 2021): The essential features of the dangerous climate change game are simulated in a ‘nutshell’: ‘Will a group of people reach a fixed target sum through successive individual monetary contributions, when they know they will lose all their remaining money with a certain probability if they fail to reach the target sum for averting simulated dangerous climate change? ‘ (Milinski et al., 2008).

We test with student volunteers whether a polycentric approach helps mitigate simulated climate change better than an approach based solely on more ‘global’ cooperation (see Methods, instructions for participants in SI). In short, the approach compares the success of groups of 9 players each to assemble the ‘global’ target sum with that of such groups subdivided in thee subgroups each assembling their subgroup target; the subgroup targets need to add up to the group target to pay off ‘globally’. Further treatments test Ostrom’s idea that incentives of refunded saved energy that subgroups are offered or sanctions of subgroups by other subgroups may help groups meeting the ‘global’ target. Although we follow the tradition of experimental economics (e.g., Smith, 1986) using small groups mimicking reality, results need to be discussed with caution when compared to reality. We provide a proof of principle, meaning that the principle is shown to be correct, although in reality it might be affected by many influences that have been controlled for in the laboratory. Vernon Smith discusses how economic experiments reflect reality (Smith, 2002).

Dangerous climate change (Schneider, 2001) might occur when a certain temperature threshold is passed (COP21, 2015; Meinshausen et al., 2009; Peters et al., 2013). Such threshold-passing is averted in our game if the nine participants of a group collectively reach the specified target sum of €180 after 10 rounds, through individual contributions per round to a climate account, from their personal account that carries an initial endowment of €40. All nine members of a group are paid out anonymously the money left in their personal account after the game. If they fail to meet the target, they lose all their money with a probability of 90%.

Though threshold variation occurs in reality (Barret and Dannenberg, 2012), we use a fixed threshold mimicking the 2 °C threshold (Randalls, 2010) in our experiments. This is what humans have been told and thus may have in mind when deciding to invest in mitigation in the real game. To increase external validity, we informed participants that all the money they paid into the climate account would be used to purchase an advertisement on ‘how to mitigate climate change’ in each of four German newspapers (see Supplementary Information).

**Fig. 1:**
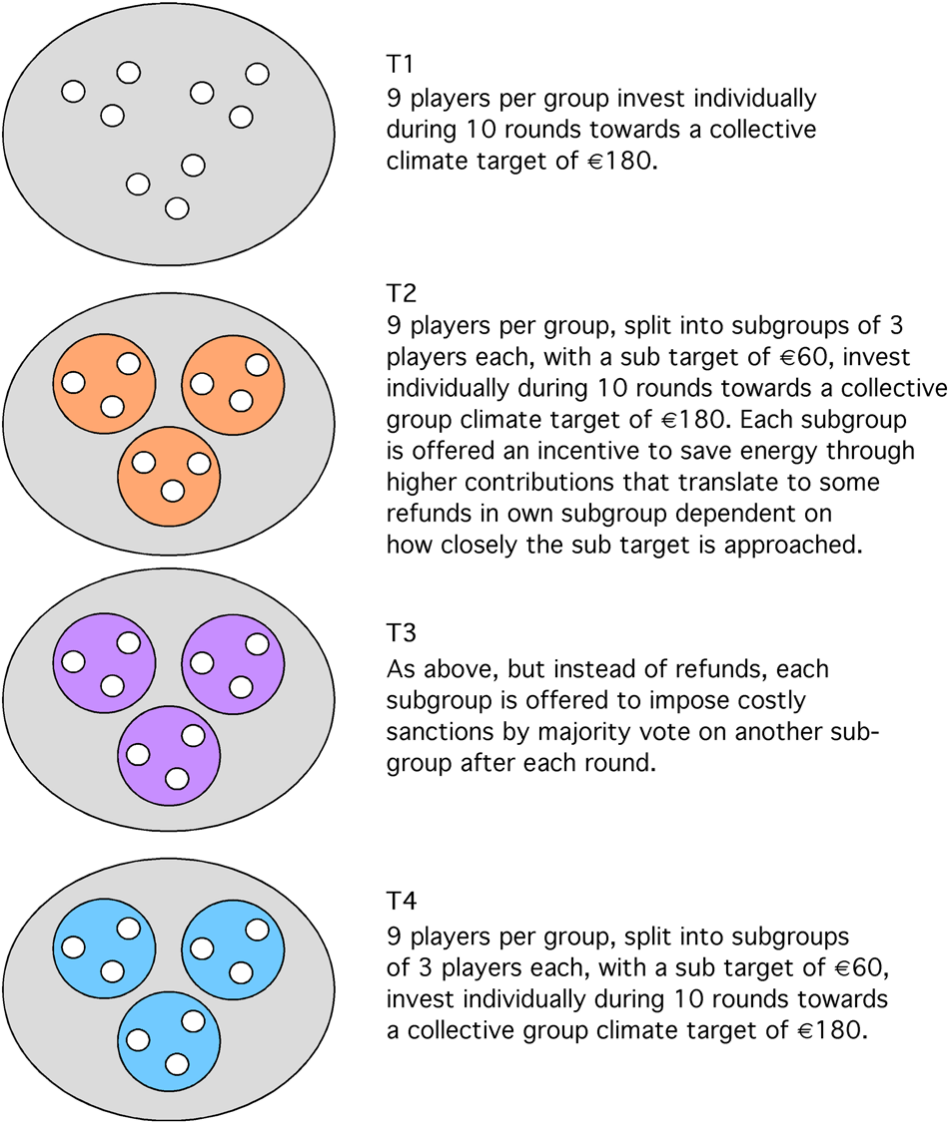
Description of experimental treatments. T1: 9 players of a group invest individually. T2: groups are split into subgroups of 3 players each, offered an incentive to save energy. T3: each subgroup is offered to impose costly sanctions on another subgroup. T4: groups are split into subgroups of 3 players each, no incentive is offered, no opportunity to impose costly sanctions is offered.

We use groups of nine volunteers in a collective-risk social dilemma investing individually over 10 rounds towards a group target, either in a single group of nine (treatment T1), or the nine players are subdivided in three subgroups of three players each (treatment T2, T3, T4) (Fig. 1). In T2 each player has the incentive to gain an extra monetary reward if her subgroup of three has approached the sub-target of €60 after ten rounds, starting refund at €52 reached and increasing it until €60 reached (Fig. 1, see Methods for procedure). The players are told that contributions simulate investment in an energy-saving block heat and power plant for the community that starts saving costs with a sub-target approached (Ostrom, 2009a, 2012). The extra monetary refund is paid out after the game irrespective of whether the final global target sum of €180 has been assembled by the nine players. The comparison between T1 and T2 tests Ostrom’s original idea—polycentricity combined with incentives. Will any of the subgroups in T2 reach the subgroup target of €60 with higher probability than *post-hoc* randomly formed subgroups in T1, and will the groups of nine players reach the ‘global’ target of €180 with higher probability in T2 than in T1?

With treatment T3 (see Methods, Fig. 1) we test the predictions of a dynamic evolutionary model (Vasconcelos et al., 2013; Pacheco et al., 2014) that sanctions boost mitigation by groups in a polycentric setting. Each of the three subgroups can, at a small cost to each member, sanction another subgroup by majority vote. Each member of the sanctioned subgroup has to pay a larger fine. Each subgroup is shown the contribution of each subgroup. One or two subgroups can decide, if at least two members of a subgroup happen to agree to sanction a specific other subgroup, that has, e.g., contributed below the fair share in the last round, to sanction that subgroup.

Our treatment differs from the model assumption in one important aspect. In the model (Vasconcelos et al., 2013; Pacheco et al., 2014) each subgroup can avert climate change and gain the payoff by exceeding only its subgroup threshold. Therefore, it makes sense if group members sanction free-riders within their own subgroup. In the present study, however, subgroups can gain the payoff only when subgroups jointly invest to reach the ‘global’ target, as in reality. Will any of the subgroups in T3 reach the subgroup target of €60 with higher probability than subgroups in T4, a control without the sanctioning opportunity, and will any of the groups of nine players reach the global target of €180 with higher probability in T3 than in T4?

## Methods

### Participants and procedure

Experiments were conducted in November, December 2014, and in April, May 2015 with a total of 396 undergraduate students from the universities of Kiel, Hamburg, Köln and Greifswald, Germany. The students received a show-up fee of €10 each and participated in 44 experimental sessions with nine participants each in a computerised experiment using the software z-Tree (Fischbacher, 2007). The participants were separated by opaque partitions and each had a computer on which they received the instructions for the experiment and with which they communicated their decisions.

Participants were randomly allocated to experimental groups, were anonymous throughout the whole experiment, and made their decisions under a neutral pseudonym. Pseudonyms were moons of our solar system: Ananke, Telesto, Despina, Japetus, Kallisto, Metis, Galatea, Vestia, Leda.

### Treatments

We use groups of nine volunteers to simulate a ‘country’, either in a single group of nine (T1)—10 groups, or the nine players are subdivided in three subgroups of three players each (T2—12 groups, T3—12 groups, T4—10 groups) (Fig. 1). We use groups of 9 volunteers, to be divided in subgroups of equal size. The participants interacted in a variant of the ‘collective-risk social dilemma’ game (Milinski et al., 2008). They received an initial endowment of €40 and were asked, in each of ten rounds, to contribute €0, €2 or €4 from this endowment into a ‘climate account’. After ten rounds, the game software checked whether total contributions of all group members matched (or exceeded) the target sum of €180. If that was the case, that is each group member had paid €2 per round on average, participants received the money in their account in cash in a way that maintained the participants’ anonymity. If the collective target was not reached, participants lost their remaining money with 90% probability. All money contributed to the climate account, regardless of country success was used to publish a newspaper advertisement on ‘how to mitigate climate change’ composed by the Max Planck Institute for Meteorology (see Supplementary Information). To gain external validity, all money invested in mitigation is lost by the players, as in reality, and is now invested in the advertisements, irrespective of the success of mitigation.

In treatment T2, the nine players are subdivided in three subgroups of three players each and each player has the incentive to gain an increasing extra monetary reward of €0.50 if her subgroup of three has reached a sub-target starting at €52. Rewards increase with higher sub-targets reached after ten rounds, paid out anonymously after the game, irrespective of whether the final global target sum of €180 has been assembled by the nine players, sub-target reached after 10 rounds: €52—reward per player: €0.50 €, sub-target reached after 10 rounds: €54—reward per player: €1.00 €, sub-target reached after 10 rounds: €56—reward per player: €2.00 €, sub-target reached after 10 rounds: €58—reward per player: €4.00 €, sub-target reached after 10 rounds: €60 and more—reward per player—€8.00.

Treatment T3 is as T2 without incentives, but each of the three subgroups can costly sanction another subgroup by majority vote. Simulated dangerous climate change is averted only when the group target, not the subgroup, target is reached. If at least 2 of the 3 players of a subgroup have anonymously voted to sanction another specific subgroup, each player of the active subgroup pays €0.50 and each of the sanctioned subgroup €2.00 from his/her account ‘extra expenses’, €15 per player. Only in T3 does each player receive an additional funds called ‘extra expenses’, from which costs of sanctioning and being sanctioned are covered. Each player receives the money that has not been spent from his/her ‘extra expenses’ anonymously after ten rounds irrespective of whether the collective target of €180 has been reached. Treatment T4 is the same as T3 but without the sanctioning opportunity (Instructions to participants, see Supplementary Information).

### Statistics

All *P*-values are two-tailed. Except for regression analysis we use non-parametric statistics throughout.

## Results

### The polycentric approach and Ostrom’s incentive

**Fig. 2:**
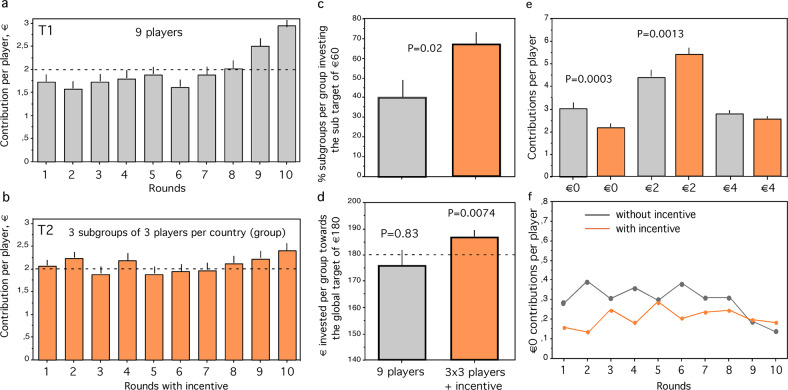
Investment into climate change mitigation. **a** Contribution per player of 9 players per group investing individually during each of 10 rounds (T1). Stippled line shows fair share of €2. **b** Contribution per player of 9 players per group subdivided into 3 fixed subgroups of 3 players investing individually in mitigation during each of 10 rounds with an incentive to save energy, benefiting own subgroup (T2). **c** Percentage of subgroups per group investing the sub-target of €60, for randomly simulated subgroups of 3 players in T1 (grey) and in T2 (orange). **d** Euros invested per group towards the global collective target of €180 (stippled line), for T1 (grey) and T2 (orange). **e** Selfish (€0), fair (€2) and generous (€4) contributions per player per round for randomly simulated subgroups investing without incentive (grey), and for fixed subgroups with incentive (orange). **f** Selfish (0€) contributions per player in each round for randomly simulated subgroups in T1 (grey) and for the fixed subgroups in T2 (orange). See text for statistics, bars indicate standard errors.

In T1 individual contributions are mostly below the fair share of €2 on average except for the last two rounds (Fig. 2a), and the target sum of €180 is not reached on average (Fig. 2d, *P* = 0.83, *z* = −0.205, *N* = 10 groups, Wilcoxon one-sample test). In T2 the groups assembled significantly more than €180 on average (Fig. 2d, *P* = 0.0074, *z* = −2.68, *N* = 12 groups, Wilcoxon one-sample test) and more than groups in T1 (*P* = 0.045, *z* = −2.005, N1 = 10, N2 = 12, Mann–Whitney *U*-test). A higher percentage of subgroups in T2 reached their sub-target of €60 than randomly formed subgroups of three players in T1 (Fig. 2c, *P* = 0.0196, *z* = −2.334, N1 = 12, N2 = 10, Mann–Whitney *U*-test).

Thus, incentives for reaching a subgroup goal repaid eventually through savings of energy produced the ‘global’ solution in the experiment, supporting Ostrom’s (Ostrom, 2009b) hypothesis. Depending on how closely subgroups approached the subgroup target of €60, repaid investments ranged from 0 to €8 per player (see Methods). In this way players in this treatment gained €6.40 ± €0.24 per player, which is much less than €20 per player to be gained on average when the overall target of €180 was contributed.

### Do incentives reduce selfish contributions and/or increase generous contributions?

Players could contribute either €0, €2 or €4 in each round. Selfish contributions (0€) were more frequent in T1 than in T2 (Fig. 2e, f; T1: 2.978 ± 0.211, mean ± SE; T2: 2.12 ± 0.158, *P* = 0.0013, N1 = 30, N2 = 36 subgroups, *z* = −3.205, Mann–Whitney *U*-test). Fair contributions per player (€2) were less frequent in T1 than in T2 (Fig. 2e; T1: 4.211 ± 0.243; T2: 5.343 ± 0.216; *P* = 0.0013. *z* = −3.225, N1 = 30, N2 = 36 subgroups, Mann–Whitney *U*-test). Generous contributions per player (4€), however, did not significantly differ between T1 (2.711 ± 0.164) and T2 (2.5 ± 0.156; *P* = 0.3182, N1 = 30, N2 = 36 subgroups, *z* = −0.998, Mann–Whitney *U*-test; Fig. 2e). Thus, polycentricity combined with incentives reduces selfish contributions, increases fair contributions but hardly affects generous contributions.

### Sanctioning does not help reach the target

**Fig. 3:**
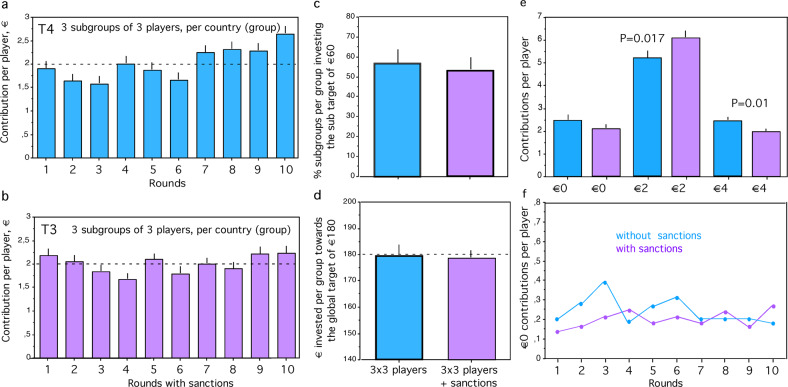
Sanctions and climate change mitigation. **a** Contribution per player of 9 players per group each subdivided into 3 fixed subgroups of 3 players investing individually in mitigation during each of 10 rounds (T4). Stippled line shows fair share of €2. **b** Contribution per player when each subgroup is offered to impose costly sanctions on another subgroup after each round (T3). **c** Percentage of subgroups per group investing the sub-target of €60, for fixed subgroups (blue) and for fixed subgroups with the opportunity to impose costly sanctions (pink) **d** Euros invested per group towards the global collective target of €180, see stippled line. **e** Selfish (€0), fair (€2) and generous (€4) contributions per player per round with fixed subgroups investing without sanction opportunity (blue), and with sanction opportunity (pink). **f** Selfish (0€) contributions per player in each round with fixed subgroups investing without sanction opportunity (blue), and with sanction opportunity (pink). See text for statistics, bars indicate standard errors.

When subgroups have the opportunity to impose sanctions on another subgroup (T3), at least two players of a subgroup have to select the same subgroup to be disciplined. Each player of the active subgroup has a cost of €0.50 and each player of the sanctioned subgroup has a cost of €2.00. Sanctioning was used extensively, 9.0 ± 0.937 per group during the 10 rounds, almost once per round. Sanctioning had, however, almost no effect on contributions per round as compared to T4 (Fig. 3a, b). Contributions per group towards the target of €180 were on average close to the target sum in T3 but not different from T4 (Fig. 3d, *P* = 0.5529. N1 = 10, N2 = 12, *z* = −0.596, Mann–Whitney *U*-test). Similarly, the percentage of subgroups per group investing the subgroup target of €60 averaged over all groups was slightly higher than 50% in T3 but not different from T4 (Fig. 3c, *P* = 0.6608, N1 = 10, N2 = 12, *z* = −0.439, Mann–Whitney *U*-test). Contributions per player increased from the first half to the second half in T4 (Fig. 3a, *P* = 0.0005, *N* = 30 subgroups, *z* = −3.463, Wilcoxon-signed-rank test) but not in T3 (Fig. 3b, *P* = 0.2939, *N* = 36 subgroups, *z* = −1.05, Wilcoxon-signed-rank test).

Selfish contributions per player (0€) did not differ significantly between T3 (2.00 ± 0.163) and T4 (2.367 ± 0.212; *P* = 0.16, *z* = −1.398, N1 = 30 subgroups, N2 = 36 subgroups, Mann–Whitney *U*-test; Fig. 3e). Fair contributions per player (€2) were more frequent in T3 (6.00 ± 0.245) than in T4 (5.178 ± 0.246; *P* = 0.0166, *z* = −2.395, Mann–Whitney *U*-test; Fig. 3e).

Generous contributions per player (4€) were significantly less frequent in T3 (1.898 ± 0.158) than in T4 (2.367 ± 9129; *P* = 0.0091, *z* = −2.606, Mann–Whitney *U*-test; Fig. 3e). Thus, overall contributions were similar with and without sanction opportunity. Polycentricity combined with a sanction opportunity hardly affects selfish contributions, increases fair contributions but decreases generous contributions compared to polycentricity without a sanction opportunity.

### The puzzle of sanctioning without disciplining

**Fig. 4:**
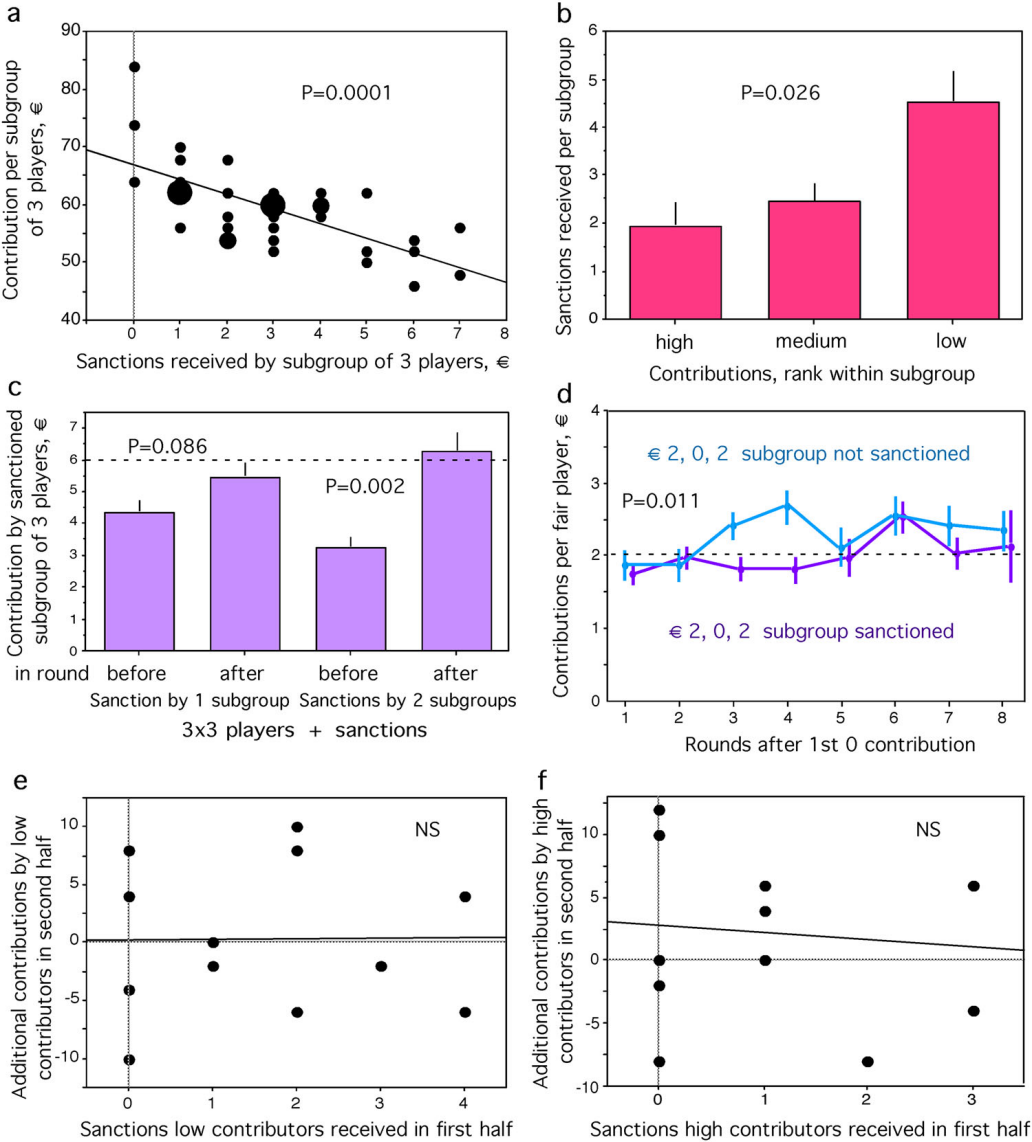
The effect of sanctions. **a** Regression of sanctions received by subgroups of 3 players on the total contribution of subgroup. Bigger dots indicate overlay. **b** Sanctions received per subgroup whose total contribution ranks either high, medium or low within its group. **c** Mean contribution by sanctioned subgroup in round before and after sanctioning by one or two subgroups, respectively. Bars indicate standard errors. Stippled line indicates the fair contribution per subgroup. **d** Contribution per player that had contributed €2 when the subgroup was sanctioned because another player had contributed €0, during rounds after the sanctioning was received (T3, pink); only first instance of sanctioning per subgroup analysed. The same analysis carried out in the treatment for the first occurring triplet of €0, €2, €2 contributions (T4, blue). Bars indicate standard errors. Stippled line indicates fair share per player. **e** Regression of sanctioned players with the lowest contribution rank in a subgroup received during the first 5 rounds on their additional contributions during the last 5 rounds of the experiment. **f** Regression of sanctioned players with the highest contribution rank in a subgroup received during the first 5 rounds on their additional contributions during the last 5 rounds of the experiment. See text for statist.

Figure 4a shows a strong correlation between the number of sanctions received by a subgroup and its contribution toward the target sum (*P* = 0.0001, *N* = 36, *r*-squared = 0.466, *F*-test = 29.723): subgroups that have contributed least have been sanctioned most. Thus, sanctioning does not equalise contributions among subgroups. The number of sanctions received is highest in the subgroups with the lowest contribution rank in a group (Fig. 4b). Average number of sanctions received differs among the three subgroup-contribution ranks per group (Fig. 4b) (*P* = 0.0258, Chi-squared = 7.316, Friedman-test).

Do subgroups react to being sanctioned? Contributions of subgroups that are sanctioned by one subgroup are unfair—they contribute below €6 per round (*P* = 0.0004, *N* = 31, *z* = −3.538, Wilcoxon one-sample test) and then increase contributions to the next round but not significantly so (*P* = 0.0863, *N* = 31, *z* = −1.715, Wilcoxon-matched-pairs test), remaining on average still below €6 (Fig. 4c). When two subgroups jointly sanction a subgroup, the disciplining effect increases significantly (*P* = 0.0021, *N* = 16, *z* = −3.081, Wilcoxon-matched pairs test) with contributions in the next round slightly above €6 on average (Fig. 4c). The disciplining effect vanishes quickly, sanctions in the first half, rounds 1 to 5, do not affect the change in contribution from the first to the second half (rounds 6 to 10) neither in contributors ranking lowest per subgroup (Fig. 4e,*P* = 0.9615, *N* = 12 subgroups, *R*-squared = 2.452E−4, *F*-test=0.002) nor in contributors ranking highest per subgroup (Fig. 4f, *P* = 0.7752, *N* = 12 subgroups, *R*-squared = 0.009, *F*-test = 0.086). Thus, sanctioning has only a short-term disciplining effect.

### Sanctioning without disciplining explained

When a subgroup is sanctioned because one player has contributed only €0, the other two players have usually contributed the fair share (€2). Those fair players contribute less during rounds after the sanctioning (T3, Fig. 4d), with only the first instance of sanctioning per subgroup analysed to avoid pseudo-replication. They contribute less (€1.972 ± 0.075) than comparable players in T4 (€2.366 ± 0.113), for the first occurring triplet of €0, €2, €2 contributions (*P* = 0.01, *z* = −2.558, N1 = 45, N2 = 54, Mann–Whitney *U*-test). Thus, the fair contributors react to their subgroup being sanctioned, which is unfair to them. Increased contributions by sanctioned free riders are compensated by the reduced generosity of co-sanctioned fair players, nullifying any sanctioning effect at the entire-group level (Fig. 3d). Sanctioning had a cost of €1.486 ± 0.067 and being sanctioned of € 5.944 ± 0.377 per player overall, a sum of €7.40 on average, with a range of €0.50 to €16, which reduces the extra account of €15 to €7.60 ± 0.38, similar to the extra payoff of €6.40 ± 0.24, with a range of €0 to €8, gained per player in T2.

## Discussion and conclusions

‘The problem of averting massive climate change—or a global ‘public bad’—would be a global ‘public good’’ (Sandler, 2004). Billions of actors affect the global atmosphere. All would benefit from reducing greenhouse gas emissions, but the problem is that an actor benefits whether or not he or she pays any of the mitigation costs. ‘Trying to solve the problem of providing a public good is a classic collective-action dilemma—and potentially the largest dilemma the world has ever knowingly faced‘ (Ostrom, 2014). ‘No central authority exists at the global level making authoritative decisions about payments for energy use and investments in new technologies—and enforcing these decisions‘ (Ostrom, 2014). ‘It is much easier to craft solutions for collective-action problems related to smaller-scale common pool resources than for the global commons‘ (Ostrom, 2014). Ostrom hypothesised that the successful polycentric solutions would also scale up to the global commons (Ostrom, 2014; Ostrom, 2010; Dietz et al., 2003).

We find support for Ostrom’s hypothesis in an economic game by comparing climate mitigation at the ‘global’ level with that in a world subdivided into smaller communities. In our game, the groups of volunteers are subdivided into subgroups and, according to Ostrom’s suggestion, are provided with an incentive of repaid saved energy the closer their subgroup approaches a sub-target. Polycentric groups with incentives for subgroups reach the global target significantly more often than groups without subdivision and without incentives for subgroups. With polycentricity and incentives, subjects choose less often zero-contributions and more often the fair-share contribution. This polycentric approach is about 30 percent more successful in reaching the global target at the entire-group level.

Contrary to the predictions of a dynamic evolutionary model (Vasconcelos et al., 2013; Pacheco et al., 2014) we do not find that sanctioning, even when frequent, helps avoiding simulated dangerous climate change. There is a difference between model and experimental assumptions. In the model, each subgroup can avert climate change by exceeding only its subgroup threshold. Therefore, it makes sense in the model if group members sanction free-riders within their own subgroup. In the present study, subgroups can avert climate change only when subgroups jointly invest to exceed the ‘global’ threshold, as in reality. It does not help if the Netherlands reach their group target whereas Germany fails—the climate will be the same for both countries (Milinski, 2014). When subgroups sanction other subgroups for a round of contributing below fair share, having a free-rider among them, the free-rider is disciplined. However, subgroup sanctioning also hits the fair contributors and provokes them to reduce their further share neutralizing the positive effect of sanctioning. Sanctions that are perceived as unfair can deter altruistic behaviour (Fehr and Rockenbach, 2003). An experiment on sanctions applied to an entire group on account of a single free-rider found that such collective sanctions are ineffective (Chapkovski, 2021), corroborating our findings.

Ultimately, climate change is caused by human behaviour. Indeed, ‘one lesson from decades of research in psychology is that human behaviour is strongly embedded in communities, be it neighbourhoods, information groups, or networks of friends or professionals. These social networks may often help to convey clues for change, remove barriers to change, and provide the norms and sometimes support needed for sustainable behavioural change‘ (Huckelba and Van Lange, 2020). Humans use their toolbox for decision-making when identifying the most useful option on their own behalf (Andrews et al., 2018). The toolbox can be increased by integrating psychological concepts into feasible interventions to reduce greenhouse gas emissions (Van Lange et al., 2018; Nielsen et al., 2021) at the level of small communities.

The overall landscape of climate governance has started to exhibit some of the characteristics of polycentricity foreseen by Ostrom (2010), ‘that is, more diverse, multi-levelled, and with a much greater emphasis on bottom-up initiatives‘ (Jordan et al., 2015). Research has shown that the advent of new forms of climate governance has made the overall landscape more polycentric: ‘Spanning many spatial levels and working through many modes and domains of actions. This pattern bears out many, but not all, of Ostrom’s predictions‘ (Jordan et al., 2015). A polycentric approach to climate governance has been argued to ‘provide the best chance we have of accelerating progress toward global climate stabilisation’ (Cole, 2015). We have provided experimental evidence for this reasoning using methods of experimental economics. We find, though at a small experimental scale, that polycentric settings with benefits for subgroups can in principle, as we conclude with caution, help protect a global common good such as a stable global climate. By contrast, sanctioning free-riding subgroups has proved ineffective.

How can we experimentally study interactions at larger scales, such as states collaborating in mitigating climate change, even though there is always the risk of free-riding of some states on the investments of others as Ostrom mentions in several places? It is possible that some states can enforce higher investments from other states, as a simulation of climate conferences has shown in an economic experiment (Milinski et al., 2016). Selfish players were preferentially elected as representatives, who turned out to be extortioners (c.f. Press and Dyson, 2012). Their steadfast strategies enforce cooperation from other representatives who compensate completely the deficit caused by extortionate players so that mitigation was successful—not a nice but successful interaction among simulated ‘states’.

## Supplementary information

Supplementary Meterial

## Data Availability

Data are available from https://hdl.handle.net/21.11116/0000-000B-5C25-5.
