# Supplementary material for: Economic experiments support Ostrom’s polycentric approach to mitigating climate change
Source: Humanit Soc Sci Commun. 2022 Dec 10;9(1):442. doi: 10.1057/s41599-022-01436-6 (PMC9735278; doi:10.1057/s41599-022-01436-6)
Supplement: Supplementary file 1 — Supplementary Meterial [file 41599_2022_1436_MOESM1_ESM.pdf]

Supplementary Information for

Economic experiments support Ostrom's polycentric approach to mitigating climate change

1. **Advertisement in daily newspapers of those University cities where experiments were performed**
2. **Instructions for participants**

1. Advertisement in daily newspapers of those University cities where experiments were performed

MAX-PLANCK-INSTITUT
FÜR METEOROLOGIE

Professor Jochem Marotzke, Direktor am Max-Planck-Institut für Meteorologie:

"**Der Mensch hat das globale Klima bereits nachweislich geändert,** und weitere, wesentlich größere Änderungen sind für dieses Jahrhundert zu erwarten. Der Ausstoß von CO_2 und anderen Treibhausgasen wird die globalen Erwärmung weiter verstärken. Als Folge müssen wir mit häufigerem Auftreten von Klima- und Wetterextremen rechnen. Hitzewellen wie 2003 in Europa, mit über 15.000 Todesfällen allein in Frankreich, werden häufiger auftreten. Sowohl Dürren als auch Extremniederschläge und Überschwemmungen werden zunehmen. Der Meeresspiegel wird ansteigen, und dadurch die Gefahr extremer Sturmfluten."

"Einige künftige Klimafolgen des menschengemachten CO_2-Aus- stoßes, wie eine gewisse Erwärmung und ein Anstieg des Meeresspiegels, sind bereits nicht mehr abwendbar, und die Menschheit muss sich diesen Veränderungen anpassen. Andere Folgen sind durch eine Verringerung des CO_2-Ausstoßes vielleicht abwendbar. Maßnahmen im täglichen Leben können zum Klimaschutz beitragen:

- Dreiviertel des Energieverbrauchs in Privathaushalten erfolgt durch Raumheizung. Durch eine leichte Verringerung der Raumtempertur im Winter lässt sich viel Energie einsparen.
- Im Verkehr besteht eine Energiesparmöglichkeit durch eine stärkere Nutzung öffentlicher Verkehrsmittel statt des Privat-PKWs.
- Der verstärkte Einsatz regenerativer Energiequellen trägt zu einer Verminderung des CO_2 -Ausstoßes bei."

Verantwortlich für den Text: Professor Jochem Marotzke, Max-Planck-Institut für Meteorologie, Hamburg. Diese Anzeige wurde durch Spenden finanziert. Studierende der Universitäten Greifswald, Hamburg, Kiel und Köln nahmen im November und Dezember 2014 und im April und Mai 2015 an einem „Klimaspiel" teil, das die Bereitschaft der Teilnehmer erforschte, eigenes Geld für den Klimaschutz auszugeben. Das „Klimaspiel wurde durch Professor Manfred Milinski, Max-Planck-Institut für Evolutionsbiologie, Plön, in Kooperation durchge-führt.

Hamburger Abendblatt, December 18, 2021, € 2377,14 VAT included

Kieler Nachrichten, December 18, 2021, € 1.992, 82 VAT included

Kölner Stadt-Anzeiger / Kölner Rundschau, December 18, 2021, € 3996,02 VAT included

Ostsee-Zeitung (Greifswald), December 18., 2021, € 1995,87 VAT included

Total €10.361,85 VAT included

2. Instructions for participants

Instructions for T1

Welcome to this experiment in which you can earn money!

At the start of the experiment, 40 Euros as a personal endowment will be credited to your account. In the course of the game, you can decide whether money from your endowment will be invested or not. In addition, you receive10 Euros for your participation in this game as a “show up fee”. You will receive this money anonymously in cash after the game irrespective of its final results. These 10 Euros cannot be used for investments during the game. You decide anonymously. To ensure this, the computer assigns a pseudonym to you, visible at the bottom left of the screen. These pseudonyms are names of moons in our solar system (Ananke, Telesto, Despina, Japetus, Kallisto, Metis, Galatea, Vestia, Leda).

For the experiment to be successful you are not allowed to talk to the other participants or make yourself noticeable to them in any way.

All information obtained during the course of the game must be confirmed by clicking NEXT.

After reading this text completely, please confirm by clicking NEXT.

In the course of this experiment, you will play **exactly 10 climate rounds**.

In each of these rounds you can invest in an attempt to protect climate and to avoid dangerous climate change. One of the consequences of dangerous climate change will be serious economic loss, which is simulated in this game.

In each round of the game all nine players will be asked simultaneously:

"How much do you want to invest in climate protection?"

(possible answers: €0, €2 or €4)

When every player has made his decision all ten choices are displayed simultaneously on all nine laptops. Afterwards all contributions will be credited to an account for climate protection.

Of the money that has eventually been invested in climate protection, we will place an advertisement in your local newspaper. This advert will give general information about simple methods for climate protection - methods that everybody can implement without much effort to protect our climate and to assist in avoiding dangerous climate change. The more money we collect the larger and more conspicuous the advertisement will be. If this promotion is successful, sponsors for international advertising campaigns could be eventually mobilized.

After reading this text completely, please confirm by clicking NEXT.

Professor Jochem Marotzke, Director of the Max-Planck-Institute for Meteorology in Hamburg will provide expertise about the state of the climate for the advertising copy. He will also give some recommendations on how to abate CO_2-emissions and how the climate can be protected:

For example, energy can be saved by decreasing ambient room temperature or by the use of public transportation instead of private cars. Furthermore, the increased use of renewable energy sources contributes to greenhouse gas abatement.

After reading this text completely, please confirm by clicking NEXT

Continued: climate round

After each round the decisions of all players are displayed.

Example:

Four players have decided to invest into climate protection. Two of them paid €2 and two further players paid €4.

pseudonym	decision	change of account
Kalypso	yes	-2
Triton	no	0
Portia	no	0
Sinope	yes	-4
Carpo	yes	-2
Iocaste	yes	-4

Kore	no	0
Phobos	no	0
Deimos	no	0

In total 12 euros were paid for climate protection and thus credited to the **climate account**.

After reading this text completely, please confirm by clicking NEXT

The end of the game

At the end of the game (after exactly 10 rounds) the computer compares the climate account with the threshold amount of €180. This threshold amount has to be reached to avoid dangerous climate change. It is reached if every player has paid on average €2 per round for climate protection. If the amount of €180 has been assembled in the climate account, each player will obtain whatever is left over in his account. The money will be paid out in cash anonymously under your pseudonym.

If the threshold amount of €180 has **not** been assembled in the climate account dangerous climate change occurs with a probability of **90 %**

thus in 9 out of 10 cases

followed by serious economic losses. **This is the probability of losing all the money in your account and none of the nine players receives a payment.**

After reading this text completely, please confirm by clicking NEXT.

The experiment will start now

After reading this text completely, please confirm by clicking NEXT.

Instructions for T2

Welcome to this experiment in which you can earn money!

At the start of the experiment, 40 Euros as a personal endowment will be credited to your account. In the course of the game, you can decide whether money from your endowment will be invested or not. In addition, you receive10 Euros for your participation in this game as a “show up fee”. You will receive this money anonymously in cash after the game irrespective of its final results. These 10 Euros cannot be used for investments during the game. You decide anonymously. To ensure this, the computer assigns a pseudonym to you, visible at the bottom left of the screen. These pseudonyms are names of moons in our solar system (Ananke, Telesto, Despina, Japetus, Kallisto, Metis, Galatea, Vestia, Leda).

You are together with two other players in a group (AnTeDe, JaKaMe or GaVeLe). The composition of your group is constant for the whole game. The three groups act independently but simultaneously.

For the experiment to be successful you are not allowed to talk to the other participants or make yourself noticeable to them in any way.

All information obtained during the course of the game must confirmed by clicking NEXT.

After reading this text completely, please confirm by clicking NEXT.

In the course of this experiment, you will play **exactly 10 climate rounds**.

In each of these rounds you can invest in an attempt to protect the climate and to avoid dangerous climate change. One of the consequences of dangerous climate change will be serious economic loss, which is simulated in this game.

In each round of the game all nine players will be asked simultaneously:

"How much do you want to invest in climate protection?"

(possible answers: €0, €2 or €4)

When every player has made his decision the choices of all team mates of your own group and the total deposits of the other groups are displayed. Afterwards all contributions will be credited to an account for climate protection.

Of the money that has eventually been invested in climate protection, we will place an advertisement in your local newspaper. This advert will give general information about simple methods for climate protection - methods that everybody can implement without much effort to protect our climate and to assist in avoiding dangerous climate change. The more money we collect the larger and more conspicuous the advertisement will be. If this promotion is successful, sponsors for international advertising campaigns could be eventually mobilized.

After reading this text completely, please confirm by clicking NEXT.

Professor Jochem Marotzke, Director of the Max-Planck-Institute for Meteorology in Hamburg will provide expertise about the state of the climate for the advertising copy. He will also give some recommendations on how to abate CO_2-emissions and how the climate can be protected:
For example, energy can be saved by decreasing ambient room temperature or by the use of public transportation instead of private cars. Furthermore, the increased use of renewable energy sources contributes to greenhouse gas abatement.

After reading this text completely, please confirm by clicking NEXT

After each round the choices of all teammates of your own group and the total deposit of each of the other groups is displayed.

Example:

In this example you are a player of group KaTriPo. Here two players of group KaTriPo have decided to invest into climate protection. Kalypso paid €2, Portia paid €4, Triton did not contribute anything.

pseudonym	decision	change of account

Kalypso	yes	-2
Triton	no	0
Portia	yes	-4

Your group KaTriPo allocated in total €6 to the climate account. Group SiCaLo allocated in total €0 to the climate account, group KoPhoDe allocated in total €0 to the climate account.

After reading this text completely, please confirm by clicking NEXT

The end of the game

At the end of the game (after exactly 10 rounds) the computer compares the climate account with the threshold amount of €180. This threshold amount has to be reached collectively by the three groups to avoid dangerous climate change. It is reached if each paid on average €60 to the climate account. This corresponds to €2 paid on average per player per round or on average to €6 per group per round.

If the amount of €180 has been assembled in the climate account, each player will obtain whatever is left over in his account. The money will be paid out in cash anonymously under your pseudonym.

If the threshold amount of €180 has **not** been assembled in the climate account dangerous climate change occurs with a probability of **90 %**

thus in 9 out of 10 cases

followed by serious economic losses. **This is the probability of losing all the**

money in your account and none of the nine players receives a payment.

After reading this text completely, please confirm by clicking NEXT.

Refund

Each player of a group that has paid at least €52 to the climate account until the end of the game, irrespective of whether the final global target-sum of €180 has been assembled by the nine players, receives a refund. Starting with a refund of €0.50 when €52 have been paid, this refund increases exponentially to up to €8.00 (52 - €0.50, 54- €1.00, 56 - €2.00, 58 - €2.00, 60 and more - €8.00).

For example, imagine a community, which is going to build an energy saving block heat and power plant for its heat supply. This block heat and power plant is environmentally friendly, because its energy consumption and thus its CO_2 production is lower compared to a conventional power plant. To build he block heat and power plant, the citizens of the community need to invest money. As soon as the new power plant is in action, the heating costs for the citizens decrease and they save money. Simultaneously the community mitigates climate change. The refund that each player of a group receives after round 10 if at least €52 have been paid to the climate account corresponds to saving heating costs.

After reading this text completely, please confirm by clicking NEXT.

The experiment will start now

After reading this text completely, please confirm by clicking NEXT.

Instructions for T3

Welcome to this experiment in which you can earn money!

At the start of the experiment, 40 Euros as a personal endowment will be credited to your account. In the course of the game, you can decide whether money from your endowment will be invested or not. In addition, you receive €15, credited to a second account, “other expenses”. Furthermore, you receive 10 Euros for your participation in this game as a “show up fee”. You will receive this money anonymously in cash after the game irrespective of its final results. These 10 Euros cannot be used for investments during the game. You decide anonymously. To ensure this, the computer assigns a pseudonym to you, visible at the bottom left of the screen. These pseudonyms are names of moons in our solar system (Ananke, Telesto, Despina, Japetus, Kallisto, Metis, Galatea, Vestia, Leda).

You are together with two other players in a group (AnTeDe, JaKaMe or GaVeLe). The composition of your group is constant for the whole game. The three groups act independently but simultaneously.

For the experiment to be successful you are not allowed to talk to the other participants or make yourself noticeable to them in any way.

All information obtained during the course of the game must be confirmed by clicking NEXT.

After reading this text completely, please confirm by clicking NEXT.

In the course of this experiment, you will play **exactly 10 climate rounds**.

In each of these rounds you can invest in an attempt to protect the climate and to avoid dangerous climate change. One of the consequences of dangerous climate change will be serious economic loss, which is simulated in this game.

In each round of the game all 10 players will be asked simultaneously:

"How much do you want to invest in climate protection?"

(possible answers: €0, €2 or €4)

When every player has made his decision the choices of all teammates of your own group and the total deposit of each of the other groups is displayed. Afterwards all contributions will be credited to an account for climate protection.

Of the money that has eventually been invested in climate protection, we will place an advertisement in your local newspaper. This advert will give general information about simple methods for climate protection - methods that everybody can implement without much effort to protect our climate and to assist in avoiding dangerous climate change. The more money we collect the larger and more conspicuous the advertisement will be. If this promotion is successful, sponsors for international advertising campaigns could be eventually mobilized.

After reading this text completely, please confirm by clicking NEXT.

Professor Jochem Marotzke, Director of the Max-Planck-Institute for

Meteorology in Hamburg will provide expertise about the state of the climate for the advertising copy. He will also give some recommendations on how to abate CO_2-emissions and how the climate can be protected:

For example, energy can be saved by decreasing ambient room temperature or by the use of public transportation instead of private cars. Furthermore, the increased use of renewable energy sources contributes to greenhouse gas abatement.

After reading this text completely, please confirm by clicking NEXT

After each round the choices of all teammates of own group and the total deposit of each of the other groups is displayed.

Example:

In this example you are a player of group KaTriPo. Here two players of group KaTriPo have decided to invest into climate protection. Kalypso paid €2, Portia paid €4, Triton did not contribute anything.

pseudonym	decision	change of account
Kalypso	yes	-2
Triton	no	0
Portia	yes	-4

Your group KaTriPo allocated in total €6 to the climate account. Group SiCaLo allocated in total €0 to the climate account, group KoPhoDe allocated in total €0 to the climate account.

After reading this text completely, please confirm by clicking NEXT

Imposing sanctions on other groups

In rounds 1 to 9 you can decide after each round whether you want to impose sanctions to other groups. However, this works only if at least one additional member of your group wants to sanction the same group. This is a majority vote. If the majority of a group decides to sanction a group, each member of the active group has a cost of €0.50. This money is taken from your account “extra expenses”. From each member of the sanctioned group €2 are taken from his account “extra expenses”. Since this is a democratic decision, each member of a sanctioning group who wants to sanction another group or who does not want to sanction has to pay €0.50. In case your account “extra expenses” becomes negative it will remain at 0.

After reading this text completely, please confirm by clicking NEXT

After decisions to sanction or not to sanction have been made, each player is shown the decisions of the members of her group and those of the other groups.

In the example group KaTriPo has sanctioned group SiCaLo. Therefore €0.50 are taken from each member of group KaTriPo. Group SiCaLo has been sanctioned and €2 are taken from each member of this group. Group KoPhoDe has not been

sanctioned and did not sanction any group. No money was thus taken from members of this group.

Actions in your group KaTriPo

member	group	sanction on group	account “extra expenses”
Kalypso	KaTriPo	SiCaIo	-0.50
Triton	KaTriPo	none	-0.50
Portia	KaTriPo	SiCaLo	-0.50
Sinope	SiCalo	none	-2.00
Carpo	SiCaLo	none	-2.00
Iocaste	SiCaLo	none	-2.00
Kore	KoPhoDe	SiCaLo	0.00
Phobos	KoPhoDe	KaTriPo	0.00
Deimos	KoPhoDe	none	0.00

Group KaTriPo has sanctioneed in this round group SiCaIo. Group SiCaIo did not sanction any group in this round. Group KoPhoDe did not sanction any group in this round.

After reading this text completely, please confirm by clicking NEXT

The end of the game

At the end of the game (after exactly 10 rounds) the computer compares the climate account with the threshold amount of €180. This threshold amount has to be reached collectively by the three groups to avoid dangerous climate change. It is reached if each paid on average €60 to the climate account. This corresponds to €2 paid on average per player per round or on average to €6 per group per round.

If the amount of €180 has been assembled in the climate account, each player will obtain whatever is left over in his account. The money will be paid out in cash anonymously under your pseudonym.

If the threshold amount of €180 has **not** been assembled in the climate account dangerous climate change occurs with a probability of **90 %**

thus in 9 out of 10 cases

followed by serious economic losses. **This is the probability of losing all the money in your account and none of the nine players receives a payment.**

After reading this text completely, please confirm by clicking NEXT.

The experiment will start now

After reading this text completely, please confirm by clicking NEXT.

Instructions for T4

Welcome to this experiment in which you can earn money!

At the start of the experiment, 40 Euros as a personal endowment will be credited to your account. In the course of the game, you can decide whether money from your endowment will be invested or not. In addition, you receive10 Euros for your participation in this game as a "show up fee". You will receive this money anonymously in cash after the game irrespective of its final results. These 10 Euros cannot be used for investments during the game. You decide anonymously. To ensure this, the computer assigns a pseudonym to you, visible at the bottom left of the screen. These pseudonyms are names of moons in our solar system (Ananke, Telesto, Despina, Japetus, Kallisto, Metis, Galatea, Vestia, Leda).

You are together with two other players in a group (AnTeDe, JaKaMe or GaVeLe). The composition of your group is constant for the whole game. The three groups act independently but simultaneously.

For the experiment to be successful you are not allowed to talk to the other participants or make yourself noticeable to them in any way.

All information obtained during the course of the game must be confirmed by clicking NEXT.

After reading this text completely, please confirm by clicking NEXT.

In the course of this experiment, you will play **exactly 10 climate rounds**.

In each of these rounds you can invest in an attempt to protect the climate and to

avoid dangerous climate change. One of the consequences of dangerous climate change will be serious economic loss, which is simulated in this game.

In each round of the game all 10 players will be asked simultaneously:

"How much do you want to invest in climate protection?"

(possible answers: €0, €2 or €4)

When every player has made his decision the choices of all teammates of your own group and the total deposit of each of the other groups is displayed. Afterwards all contributions will be credited to an account for climate protection.

Of the money that has eventually been invested in climate protection, we will place an advertisement in your local newspaper. This advert will give general information about simple methods for climate protection - methods that everybody can implement without much effort to protect our climate and to assist in avoiding dangerous climate change. The more money we collect the larger and more conspicuous the advertisement will be. If this promotion is successful, sponsors for international advertising campaigns could be eventually mobilized.

After reading this text completely, please confirm by clicking NEXT.

Professor Jochem Marotzke, Director of the Max-Planck-Institute for Meteorology in Hamburg will provide expertise about the state of the climate for the advertising copy. He will also give some recommendations on how to abate CO_2-emissions and how the climate can be protected:

For example, energy can be saved by decreasing ambient room temperature or by the use of public transportation instead of private cars. Furthermore, the increased use of renewable energy sources contributes to greenhouse gas abatement.

After reading this text completely, please confirm by clicking NEXT

After each round the choices of all teammates of own group and the total deposit of each of the other groups is displayed.

Example:

In this example you are a player of group KaTriPo. Here two players of group KaTriPo have decided to invest into climate protection. Kalypso paid €2, Portia paid €4, Triton did not contribute anything.

pseudonym	decision	change of account
Kalypso	yes	-2
Triton	no	0
Portia	yes	-4

Your group KaTriPo allocated in total €6 to the climate account. Group SiCaLo

allocated in total €0 to the climate account, group KoPhoDe allocated in total €0 to the climate account.

After reading this text completely, please confirm by clicking NEXT

The end of the game

At the end of the game (after exactly 10 rounds) the computer compares the climate account with the threshold amount of €180. This threshold amount has to be reached collectively by the three groups to avoid dangerous climate change. It is reached if each paid on average €60 to the climate account. This corresponds to €2 paid on average per player per round or on average to €6 per group per round.

If the amount of €180 has been assembled in the climate account, each player will obtain whatever is left over in his account. The money will be paid out in cash anonymously under your pseudonym.

If the threshold amount of €180 has **not** been assembled in the climate account dangerous climate change occurs with a probability of **90 %**

thus in 9 out of 10 cases

followed by serious economic losses. **This is the probability of losing all the money in your account and none of the nine players receives a payment.**

After reading this text completely, please confirm by clicking NEXT.

The experiment will start now

After reading this text completely, please confirm by clicking NEXT.
